# PPG2ABP: Translating Photoplethysmogram (PPG) Signals to Arterial Blood Pressure (ABP) Waveforms

**DOI:** 10.3390/bioengineering9110692

**Published:** 2022-11-15

**Authors:** Nabil Ibtehaz, Sakib Mahmud, Muhammad E. H. Chowdhury, Amith Khandakar, Muhammad Salman Khan, Mohamed Arselene Ayari, Anas M. Tahir, M. Sohel Rahman

**Affiliations:** 1 Department of Computer Science, Purdue University, West Lafayette, IN 47907, USA; 2 Department of Electrical Engineering, Qatar University, Doha 2713, Qatar; 3 Department of Civil and Architectural Engineering, Qatar University, Doha 2713, Qatar; 4 Technology Innovation and Engineering Education Unit (TIEE), Qatar University, Doha 2713, Qatar; 5 Department of Electrical and Computer Engineering, University of British Columbia, Vancouver, BC V6T 1Z4, Canada; 6 Department of CSE, BUET, ECE Building, West Palashi, Dhaka 1205, Bangladesh

**Keywords:** blood pressure, cuff-less blood pressure, mobile health, Photoplethysmogram (PPG), regression

## Abstract

Cardiovascular diseases are one of the most severe causes of mortality, annually taking a heavy toll on lives worldwide. Continuous monitoring of blood pressure seems to be the most viable option, but this demands an invasive process, introducing several layers of complexities and reliability concerns due to non-invasive techniques not being accurate. This motivates us to develop a method to estimate the continuous arterial blood pressure (ABP) waveform through a non-invasive approach using Photoplethysmogram (PPG) signals. We explore the advantage of deep learning, as it would free us from sticking to ideally shaped PPG signals only by making handcrafted feature computation irrelevant, which is a shortcoming of the existing approaches. Thus, we present PPG2ABP, a two-stage cascaded deep learning-based method that manages to estimate the continuous ABP waveform from the input PPG signal with a mean absolute error of 4.604 mmHg, preserving the shape, magnitude, and phase in unison. However, the more astounding success of PPG2ABP turns out to be that the computed values of Diastolic Blood Pressure (DBP), Mean Arterial Pressure (MAP), and Systolic Blood Pressure (SBP) from the estimated ABP waveform outperform the existing works under several metrics (mean absolute error of 3.449 ± 6.147 mmHg, 2.310 ± 4.437 mmHg, and 5.727 ± 9.162 mmHg, respectively), despite that PPG2ABP is not explicitly trained to do so. Notably, both for DBP and MAP, we achieve Grade A in the BHS (British Hypertension Society) Standard and satisfy the AAMI (Association for the Advancement of Medical Instrumentation) standard.

## 1. Introduction

Even in today’s world of technological advances, cardiovascular disease (CVD) is one of the most menacing causes of morbidity and mortality, crippling the aging population [1]. More than 4 million people die of cardiovascular diseases every year only in Europe, and, when considering the whole world, the number of deaths exceeds 17 million [2]. Hypertension is one of the leading reasons for cardiovascular diseases. Alarmingly, only in the USA, there are around a million patients with hypertension, which covers almost one-third of the population, and, shockingly, only less than half of them try to control their blood pressure [3]. Hypertension has thus been termed a ‘Silent Killer’ due to its dormant nature that, eventually, leads to untimely death [4]. Therefore, continuous blood pressure monitoring is essential. However, owing to the lack of expert physicians, as opposed to the ever-increasing number of patients, the development of automated monitoring methods seems to be the only feasible means to confront the crisis.

Several methods capable of measuring blood pressure (BP) accurately have been introduced. However, this accuracy comes at the cost of the invasiveness of such methods, which are often cumbersome to apply. For a catheter-based approach [5], not only is the intervention of an expert required, but such procedures also cause pain to already delicate patients. Clinics nowadays rely on cuff-based methods to measure blood pressure, but those, as well, are somewhat inconvenient and intrusive and, more importantly, not suitable for continuous blood pressure monitoring [6]. Therefore, for a significant amount of time, it has been the interest of the research community to develop methods and apparatus to determine blood pressure from biomedical signals in a continuous, cuff-less, non-invasive manner [7,8,9]. Though there are some popular volume-clamp-based techniques (Finapres and CNAP) to measure BP non-invasively, they are not very accurate and reliable [10,11,12,13]. Photoplethysmography (PPG) has gained a lot of popularity recently, due to its widespread inclusion in smartwatches and fitness bands and owing to its simplicity and cheapness. The idea behind PPG is rather simple: it works based on the illumination of the skin and detection of the light absorption of skin. A PPG sensor generally comprises a Light Emitting Diode (LED) light source and a photodetector [14]; the LED emits light to the skin tissue and the photodetector keeps track of the reflected light, i.e., the degree of absorption. It has been empirically established that the amount of reflected light is proportional to the volume of blood flowing in the illuminated region [15]. Since the volume of blood is related to the speed of blood flow, which relates to the pressure exerted on the arteries, the PPG signal has been prominently used for the measurement of blood pressure [16]. Furthermore, PPG signals are also used for calculating the absorption of oxygen as well as the level of hemoglobin in blood [17] and for the diagnosis of events such as hyperemia [18].

In recent years, a plethora of academic studies have been reported to assess the state of blood pressure, using biomedical signals, mainly PPG, often in conjunction with several other ones. The primary rationale behind measuring blood pressure from PPG is the association between the speed of blood flow and blood pressure. Overall, when blood vessels are contracted, blood flows rapidly, enforcing more pressure [19]. The opposite scenario is observed when the vessels are relaxed: steady blood flow diminishes the pressure. Therefore, studies have been conducted to investigate the velocity of the blood pressure pulse, which is popularly termed Pulse Wave Velocity (PWV) [20]. Based on PWV, blood pulses require a time delay to reach the periphery of the body from the heart, which is denoted as Pulse Transit Time (PTT) [21]. Two other parameters, namely, Pulse Arrival Time (PAT) and Pre-Ejection Period (PEP), are also relevant in such analyses [16]. A lot of research has been conducted to develop mathematical models of these various parameters to infer blood pressure values [22,23,24,25,26]. While most prior works revolve around fitting the delay terms, lately, several classical machine learning-based approaches have been introduced [6,19,27,28,29,30,31,32,33,34,35,36,37,38,39,40]. These methods usually take the PPG signal, along with the Electrocardiogram (ECG) signal, in most cases, and predict the values of Diastolic Blood Pressure (DBP), Systolic Blood Pressure (SBP), and Mean Arterial Pressure (MAP). Some other studies used Deep Machine Learning-based approaches for BP prediction from PPG and/or ECG signals [40,41,42,43,44,45,46,47,48,49,50]. However, they are suffering from some limitations. Firstly, most of these methods require ECG signals alongside PPG, which may be difficult to include in wearable cuff-less systems. Secondly, some of these rely on handcrafted features to predict the BP; however, to robustly compute such features, the algorithms often demand the signals to be always of near ideal condition, which is impractical. To solve these issues, in recent years, there have been a few studies that tried to estimate or reconstruct Arterial Blood Pressure (ABP) waveforms from PPG and/or ECG signals using various deep learning techniques such as 1D segmentation [51,52,53], variational autoencoders (VAEs) [54], and CycleGAN [54].

This work presents PPG2ABP, a pioneering framework in this domain that uses purpose-built, cascaded deep learning-based 1D segmentation models for continuously estimating the waveshape of the ABP signals from only PPG signals. The first segmentation network in the PPG2ABP pipeline tries to approximate the ABP waveform, while the second network takes in the approximated ABP waveforms and refines them. Furthermore, being a deep learning-based pipeline, PPG2ABP is free from the need for handcrafted features; therefore, the requirement of signals maintaining a standard shape is not essential. Moreover, the different values of interest in the literature, i.e., SBP, DBP, and MAP, can be calculated from the estimated ABP waveform, and, even in this objective, our method outperforms most of the existing works, despite not being explicitly trained to do so. The primary contributions of this study are as follows:

- To overcome the challenges in ABP estimation, we propose PPG2ABP, which is a cascaded approach to divide this challenging task into two stages and reach a robust outcome in the end.
- The Approximation network approximates the ABP waveforms, and the Refinement network refines the outputs of the Approximation network.
- Our proposed PPG2ABP only requires PPG waveforms for ABP estimation, thus mitigating the need for ECG probes in parallel to PPG collection devices. This makes the solution simple, cost-effective, and user-friendly.
- PPG2ABP performs better than most studies in the literature while working on a large dataset.

The rest of the paper is organized as follows: Section 2 described the methodology followed by this study in detail, including a brief discussion about the dataset, data preprocessing stages, and the proposed PPG2ABP pipeline. Section 3 contains the experimental setup and comparative evaluations against the prior works using various evaluation metrics. The outcomes of the experiments and ablation studies are provided in Section 4 along with interactive visualizations. Finally, the article is concluded with Section 5.

## 2. Materials and Methods

In this section, we discuss in detail the dataset used in this study and the proposed methodology. In the methodology section, we elaborate on the data preprocessing steps and the proposed PPG2ABP pipeline.

### 2.1. Dataset

To train and evaluate our proposed algorithm, a processed subset of the Multiparameter Intelligent Monitoring in Intensive Care (MIMIC-III) dataset from the PhysioNet repository [56,57] has been used. This dataset, compiled by Kachuee et al. [58,59], is present in the University of California Irvine (UCI) Machine Learning Repository [60]. The database compiled by Kachuee et al. [58] contains simultaneous PPG, ECG, and ABP signals recorded in a clinical setup [57]. The sampling rate for all signals is 125 Hz, recorded with 8-bit digital precision. The data present in this repository are in a convenient form to analyze as raw signals as they are already pre-processed following their algorithm [58]. For the sake of convenience, Kachuee et al. [59] ignored the signal episodes with too tiny or too large blood pressure values, i.e., extreme cases. They only considered signals with (60 mmHg ≤ DBP ≤ 130 mmHg) and (80 mmHg ≤ SBP ≤ 180 mmHg). In this study, we wanted to test our algorithm on a broader range of signals, since a real-world application scenario might exhibit extremely small and high BP values. Therefore, we considered signals with DBP as low as 50 mmHg and SBP as high as 200 mmHg. The statistics of the dataset are presented in Table 1. It can be observed that SBP has a comparatively greater value of standard deviation. This extensive range is likely to cause severe difficulties when predicting SBP, as hypothesized by Kachuee et al. [59].

For our analysis, we considered signal episodes of $T_{e}$ = 8.192 s long, i.e., we estimated $T_{e}$ seconds long arterial blood pressure (ABP) waveform from PPG signals of $T_{e}$ seconds long. Picking this $T_{e}$ of 8.192 seconds (which translates to 1024 samples due to the 125 Hz sampling rate of the signals) allowed us to train a sufficiently deep neural network without being crippled by extensive computational complexity. The authors have experimented with the 8.192 s PPG signal length based on many popular SBP and DBP estimations from PPG signal research works [31,61]. The following scheme was followed. In the UCI Machine Learning Repository [60], there are 12,000 PPG, ECG, and ABP records from 942 patients [59]. The “UCI Dataset” is already a filtered and processed version of the MIMIC-III Waveform database [56]. The first three parts of the UCI dataset were combined to make the train set (75% of the dataset—9000 Records). On the other hand, part four was taken as an independent test set (25% of the dataset—3000 Records). During training, a randomly selected 20% of the training set was used for validation. In total, 127,260 episodes (segments) were created from the 12,000 records (bins). Each record was segmented into episodes (segments) where each segment length was 1024 samples (8.192 s) long. Since the records of different patients are organized sequentially in the dataset, as per the dataset provider, this way of splitting data ensures that there is no leakage between training and test sets. Furthermore, we did not omit PPG signals of sub-ideal quality; rather, the random selection process led to the inclusion of a high number of low-quality signals so that the model can learn different quality signals [62].

### 2.2. Proposed Methodology

**Figure 1:**
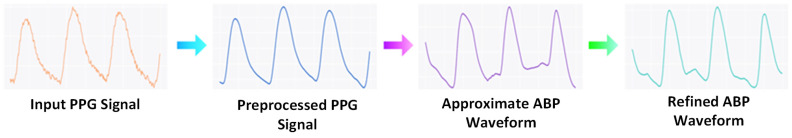
Algorithmic pipeline of PPG2ABP. PPG2ABP takes PPG segments of $T_{e}$ seconds long as the input and performs some preprocessing [58]. Next, the preprocessed signal is passed to the Approximation Network to approximate the ABP waveform. After that, the Refinement Network refines the approximated ABP waveforms. Finally, in addition to the ABP waveform, BP values such as SBP, MAP, and DBP are computed.

The proposed PPG2ABP pipeline that extracts PPG segments of $T_{e}$ seconds long performs some minimal preprocessing (e.g., filtering) operation to attenuate the irregularities and noises. Next, the filtered signal is processed using the “Approximation Network”, which approximates the ABP waveforms based on the input PPG signals. The preliminary rough estimate of ABP is further refined through the “Refinement Network”. Here, the linearly placed Approximation and Refinement networks are trained separately, maintaining the ABP waveforms as the target for both networks. PPG is the input to the Approximation network while approximated, intermediate ABP patterns are the input for the Refinement network. Finally, in addition to the estimated ABP waveform, the values of SBP, DBP, and MAP are computed in a simple manner. The PPG2ABP pipeline is depicted in Figure 1.

#### 2.2.1. Preprocessing

As mentioned earlier, for this study, we have used the signals already pre-processed by Kachuee et al. [58]. Therefore, our preprocessing steps are almost identical to theirs, except for some additional steps to prepare the dataset suitable for the deep learning pipeline. The preprocessing stage primarily involves wavelet denoising [59], which is a very popular pre-processing step to eliminate motion artifacts [63]. The wavelet transform is performed to 10 decomposition levels, with Daubechies 8 (db8) as the mother wavelet [64]. Then, the very low (0–0.25 Hz) and very high frequency (250–500 Hz) components are negated by setting the decomposition coefficients to zero, followed by soft Rigrsure thresholding [65,66]. Finally, the signal is retrieved by reconstructing the decomposition. To facilitate the training of the deep learning models, the PPG and ABP signals were independently segmented, bandpass filtered, and global min–max normalized. As mentioned earlier, the signals were segmented into episodes of 1024 data points in length following the Dirichlet rectangular windowing to facilitate training with the deep learning frameworks. A Butterworth filter with cutoff frequencies of 0.1 Hz and 30 Hz was used to filter the signals. Both PPG and ABP segments were separately normalized based on the global minimum and maximum values. All segments need to be normalized for the deep learning models as they cannot handle high amplitude signals properly, unlike classical ML models, especially when input PPG has a much lower amplitude than the target ABP signals. Training the deep learning models with normalized ABP segments will naturally produce normalized ABP segments during estimation. Now, it is required to bring back the original amplitudes of ABP signals for extracting the BP parameters (SBP and DBP). To make this process unbiased and non-leaky, we ‘min–max’ normalized the signals in the whole dataset and noted down those global min and max ABP values. After reconstruction, the ABP waveforms are denormalized using the global min–max values obtained earlier.

#### 2.2.2. Approximation Network

During this stage, the ABP signals are approximated from input PPG signals through the “Approximation Network”, which is a one-dimensional (1D), deeply supervised U-Net model. U-Net [67] had been primarily constructed using only convolutional layers for two-dimensional (2D) semantic segmentation of biomedical images. The network structure consists of symmetric pairs of encoder–decoder layers. The most innovative idea behind U-Net is the use of skip connections to preserve the spatial feature maps lost during pooling and up-sampling.

**Figure 2:**
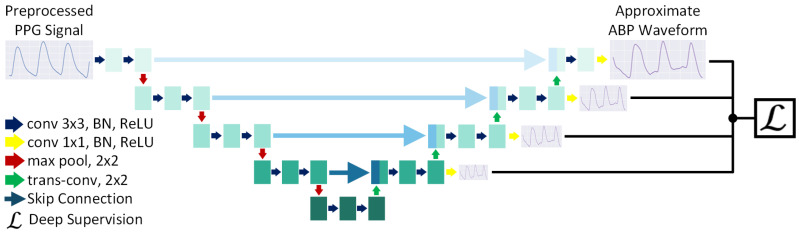
Approximation Network. PPG2ABP employs the Approximation Network to derive an approximate ABP waveform from the preprocessed PPG signal. The network is a deeply supervised one-dimensional U-Net, designed to solve regression problems.

Though the original U-Net is designed to perform semantic segmentation on images, for our purpose, we employ it to reconstruct 1D signals, which is primarily a one-to-one regression task. Therefore, the two-dimensional convolution, pooling, and upsampling operations are replaced by their one-dimensional counterparts. To produce a regression output, the final convolutional layer uses a linear activation function. Moreover, we apply deep supervision (ℒ) in our U-Net network [68]. Deep supervision is a technique proven to reduce overall errors by guiding the learning process of the hidden layers. In our deeply supervised 1D U-Net, we compute an intermediate output, which is a subsampled version of the actual output signal, before every upsampling operation in the decoder. The losses are computed with gradually declining weights as we move deeper into the model. These auxiliary losses drive the training of the hidden layers and make the final output much more superior. The diagram of the Approximation Network is presented in Figure 2.

#### 2.2.3. Refinement Network

**Figure 3:**
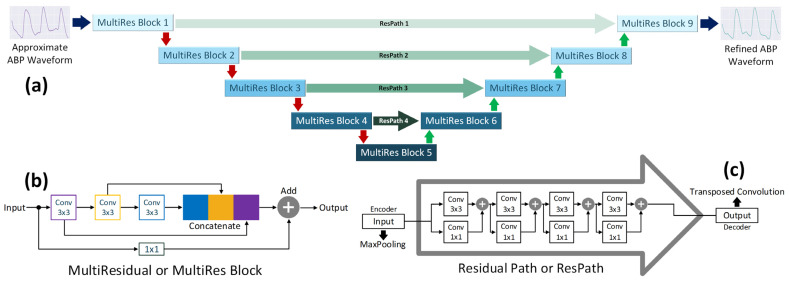
(**a**) Refinement Network. The PPG2ABP pipeline utilizes a 1D-MultiResUNet Network consisting of (**b**) MultiResidual Blocks and (**c**) Residual Paths to refine the approximate waveform constructed by the Approximation Network.

The output of the Approximation Network sometimes deviates greatly from the target. Therefore, we used an additional network, namely the ‘Refinement Network’, to refine the output of the Approximation Network. The Refinement Network is a 1D MultiResUNet model [69], an improved version of the U-Net model. The primary distinction between the two is the inclusion of the Multi-Residual, or MultiRes, blocks (Figure 3b) and the Residual, or Res, paths (Figure 3c) in the MultiResUNet model (Figure 3a). The MultiRes blocks involve a compact form of multiresolution analysis using factorized convolutions. On the other hand, Res paths impose additional convolutional operations along the shortcut connections to reduce the disparity between the feature maps of the corresponding levels of encoders and decoders.

Similar to the Approximation Network, this network comprises one-dimensional versions of convolution, pooling, and upsampling operations, and the final convolutional layer uses linear activation. However, this model is not deeply supervised. An expanded diagram of the Refinement Network is presented in Figure 3.

#### 2.2.4. BP Parameters Calculation

In addition, to construct the continuous ABP waveforms, the typical BP values of interest, namely, SBP, DBP, and MAP, can be computed by taking the max, min, and mean values of the estimated signals, respectively, as shown in Equations (1)–(3).

(1) SBP=max(ABP)

(2) DBP=min(ABP)

(3) MAP=mean(ABP)

Here, ABP is the estimated Arterial Blood Pressure waveforms from the Refinement Network.

## 3. Experiments

In this section, we reflect on various aspects of our experimental setup and insights into the ablation studies performed to determine the optimum parameters for the deep learning architectures to estimate ABP waveforms from PPG signals. For this study, we have used the Python programming language to implement the algorithms and conduct experiments. The neural network modFgitels have been developed using TensorFlow 2.0 with a KERAS frontend. Moreover, we have made the codes publicly available, which can be found in [70].

### 3.1. Selection of Models

In addition to U-Net and MultiResUNet, we also conducted some preliminary experiments using other deep-learning models. However, U-Net and MultiResUNet yielded better results. We also experimented with the combinations of U-Net and MultiResUNet for the approximation and refinement networks. It was observed that U-Net, as the refinement network, failed to reach the performance level of MultiResUNet. On the contrary, MultiResUNet, as the approximation network, performed better than U-Net, but the overall performance followed by a MultiResUNet refinement network remained quite identical. We hypothesize that though MultiResUNet is superior to U-Net and manages to obtain a much better waveform, the refinement network reaches a plateau eventually. Nonetheless, since U-Net is computationally lighter than MultiResUNet, we use U-Net and MultiResUNet as the approximation and the refinement network, respectively.

### 3.2. Selection of Loss Functions

Mean Squared Error (MSE) and Mean Absolute Error (MAE) is the most prevalently used loss function for regression. For predicted values $\;\hat{Y}=\left[{\;\hat{y}}_{1},{\;\hat{y}}_{2},{\;\hat{y}}_{3},\ldots ,{\;\hat{y}}_{n}\right]$ and the ground truth $Y=\left[y_{1},y_{2},y_{3},\ldots ,y_{n}\right],\;$they are defined in Equations (4) and (5) as follows:

(4) $$\mathrm{MSE}=\frac{\sum_{i=1}^{n}{\left(y_{i}-{\;\hat{y}}_{i}\right)}^{2}}{n}$$

(5) $$\mathrm{MAE}=\frac{\sum_{i=1}^{n}\left|y_{i}-{\;\hat{y}}_{i}\right|}{n}$$

In our experiments, we found that using MAE as the loss function of the approximation network (as opposed to MSE) significantly improves the performance. Upon inspecting samples and outputs, we developed the following rationale. Since, at the approximation network stage, we aim to obtain a rough estimate of the waveform, it suffices to put equal weights to the entire range of errors. However, MSE squares the error terms, and the bigger errors are more penalized. At this stage, we have rather little information regarding the output waveform; therefore, putting more emphasis on eliminating the bigger error terms degrades the overall performance. Thus, MAE in the approximation network stage balances all the error terms, ensuring a rough yet satisfactory projection. On the contrary, in the refinement network, we already have an approximation of the waveform. Hence, it becomes beneficial to use MSE in that stage as the larger error terms will be diminished better. The empirical evidence also supports this.

### 3.3. Effect of Number of Convolutional Filters

We have explored a pool of wider variants of the networks, comprising an increasing number of convolutional kernels or filters. From experiments, it was observed that the models with a higher number of filters performed better, which is obvious since the inclusion of additional filters would allow the model to learn and capture additional shapes and features. However, as the number of filters increases, the models become exponentially heavier and computationally more expensive. Thus, after a certain level, the improvement obtained from adding new filters is not worth the rising computational demand. Therefore, we have used a U-Net model with the number of filters as multiples of 64, i.e., (64, 128, 256, 512, 1024) and, for the MultiResUNet, we have limited the value of weight multiplier alpha (α) to 2.5, a parameter which controls the number of filters [69]. For both our approximation and refinement networks, we have used the standard configurations of the convolutional and pooling layers. In the convolutional layers, filter or kernel size was selected to be 3, while stride length was 1. Additionally, padding was used to zero-pad the intermediate outputs to keep them consistent with the input shape. On the other hand, for max-pooling layers, we used both a pool size and stride length of 2. Similarly, in the upsampling layers, a window size of 2 was selected.

### 3.4. Effect of Deep Supervision

Additionally, we have experimented with the concept of deep supervision and employed auxiliary losses during training. For both U-Net and MultiResUNet models, we have imposed additional loss functions on the outputs of the convolutional layers just before the transposed convolution (i.e., upsampling through convolution) operations. Moreover, the weights of the losses have been selected as (1, 0.9, 0.8, 0.7, 0.6), i.e., the maximum weight has been put on the final output and is gradually diminished for the premature outputs from deeper decoder layers. For the U-Net model, a dramatic improvement was observed after applying deep supervision while, for the MultiResUNet model, the improvement was minimal. Therefore, to establish a trade-off between computational effort and accuracy, deep supervision has been employed in the U-Net model (i.e., the Approximation Model) only.

### 3.5. Training Methodology

As specified in Section 3.2, MAE and MSE are used as the loss functions of the Approximation and the Refinement networks, respectively. To minimize these losses, the Adam optimizer [71] is used, which adaptively computes different learning rates for individual parameters based on the estimates of the first and second moments of the gradients. In our experiments, we used Adam with the default parameters mentioned in the original paper [71]. Each model has been trained for 100 epochs with an early stopping criterion, i.e., the patience of 20 epochs. Validation loss was used as the metric for early stopping.

### 3.6. K-Fold Cross Validation

We have performed 10-fold cross-validation using the training data, i.e., 90% of the training data is used to train the model, and the remaining 10% of the data is used for validation. This approach is repeated 10 times using different data splits, and, thus, 10 models are developed. The best-performing model is selected and is then evaluated against the independent test set.

### 3.7. Evaluation Metrics

Since we are primarily working with a regression problem, we have used Mean Absolute Error (MAE), defined in Equation (5), as our primary evaluation metric. Furthermore, we have evaluated the outcomes from the PPG2ABP pipeline using domain-specific metrics such as the British Hypertension Society (BHS) Standard, Association for the Advancement of Medical Instrumentation (AAMI) Standard, Pearson Correlation Coefficient (PCC), Bland Altman plots, etc. The details of these metrics will be presented in the following sections.

## 4. Results and Discussion

After training the PPG2ABP model, we evaluated the pipeline on the test data. The following outcomes have been derived from the evaluation of the model on the independent test set.

### 4.1. Estimating ABP Waveform

**Figure 4:**
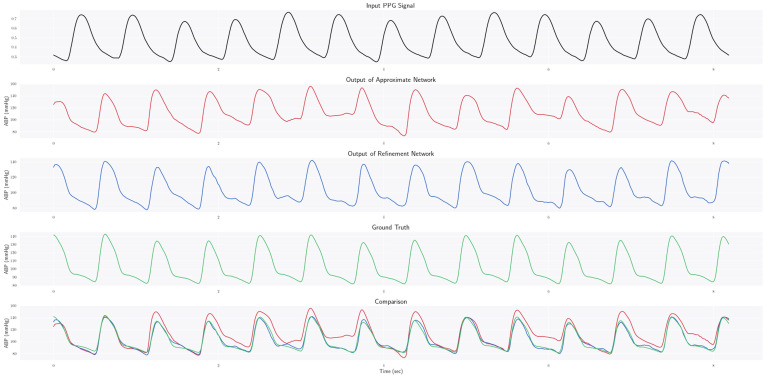
Demonstration of output from the PPG2ABP pipeline.

The primary and unique objective of this work is to transform PPG signals into the corresponding ABP waveforms. Despite some correlation between the two, as established from past studies [19,27], they are structurally quite different from each other when we consider the two waveforms. Nevertheless, the proposed PPG2ABP model manages to estimate the waveform of blood pressure by taking only the PPG signal as input. The output of the approximate network gives an overall rough estimate, which is further refined by the refinement model. Figure 4 illustrates such an example. Although the output from the Approximate Network follows the rough silhouette, it fails particularly in the rapid slope down from the peaks. However, after refining this waveform using the Refinement Network, such erroneous constructions are revised and improved, closely mimicking the ground truth. For this example, the use of the Refinement Network reduces the mean reconstruction error from 9.52 mmHg to 2.37 mmHg. The constructed waveform closely follows the ground truth waveform. Therefore, from experimental results, it is evident that PPG2ABP can translate PPG signals to corresponding blood pressure waveforms, preserving the shape, magnitude, and phase in unison. Quantitatively, the mean absolute error (MAE) of this blood pressure waveform construction is 4.604 ± 5.043 mmHg over the entire test dataset. In addition, the mean absolute error of DBP, MAP, and SBP prediction is 3.449 ± 6.147 mmHg, 2.310 ± 4.437 mmHg, and 5.727 ± 9.162 mmHg, respectively. Furthermore, previous studies have pointed out that a phase lag exists between the PPG and ABP signals of the MIMIC-III database 60, and some further processing is required to align them. However, in our generated output, we can observe that our method has been able to remarkably overcome this issue of phase lag. Indeed, this may turn out to be highly beneficial in dealing with the phase lag between the two signals in real-world applications due to the difference in Pulse Arrival Time (PAT) for recording them from two separate body locations [72]. Therefore, unlike existing works, we do not exclude irregular signals (details in the Supplementary Materials).

A sample PPG signal is shown in Figure 4, from the test data given as input. It can be observed that the output from the Approximation Network, despite roughly following the overall pattern of the ground truth, falls short in certain aspects, vividly apparent around the peaks. Furthermore, the reconstructed waveform fails to rapidly slope down from the peak regions. However, the estimation from the Refined Network seems to be more satisfactory. It can be observed that in addition to following the overall pattern of the ground truth waveform, the final estimated waveform also successfully mimics the peak regions and subsequent downward inclination. Therefore, the inclusion of the Refinement Network on top of the Approximate Network significantly improves the results, as evident from the drop in mean reconstruction error from 9.52 mmHg to 2.37 mmHg in this example.

### 4.2. BHS Standard

The British Hypertension Society (BHS) introduced a structured protocol to assess blood pressure measuring devices and methods [73]. Hence, this standard has been frequently used in the literature as a metric [6,27,58,59]. The accuracy criteria of the BHS standard appraisal methods are based on absolute error. The grades are provided by counting what percentage of the predictions on the test samples fall under 5 mmHg, 10 mmHg, and 15 mmHg absolute error for grades A, B, and C, respectively. The three grades are presented in Table 2. For an algorithm to obtain a certain grade, it must satisfy all three thresholds simultaneously. In addition, there is a grade D for algorithms failing to meet the requirements of grade C [73].

**Figure 5:**
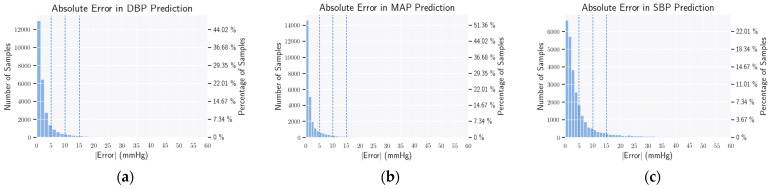
Mean absolute error histogram. Here, we present how the absolute errors of predicting DBP, SBP, and MAP of the samples are distributed. In addition, we also observe errors in how many samples lie below the 5 mmHg (**a**), 10 mmHg (**b**), and 15 mmHg (**c**) thresholds, used in the evaluation of the BHS Standard.

The absolute error of computing DBP, MAP, and SBP is presented in Figure 5. We have presented how the absolute errors of predicting DBP, SBP, and MAP of the samples are distributed, with particular focus on the error boundaries of 5 mmHg, 10 mmHg, and 15 mmHg. We have achieved grade A for both DBP and MAP, but grade B for SBP. To the best of our knowledge, the proposed algorithm is a pioneer work and one of the first that obtained a grade B in SBP prediction on a significant portion of data from the MIMIC-III dataset (more details in Section 4.5).

### 4.3. AAMI Standard

Like the BHS Standard, the Association for the Advancement of Medical Instrumentation (AAMI) Standard is another metric to evaluate blood pressure measuring devices and methods. The criterion set by the AAMI standard [74] requires the blood pressure measuring methods to have a mean error and standard deviation of less than 5 mmHg and 8 mmHg, respectively, and the minimum number of subjects under study is 85. Table 3 shows our results under the AAMI criterion.

**Figure 6:**
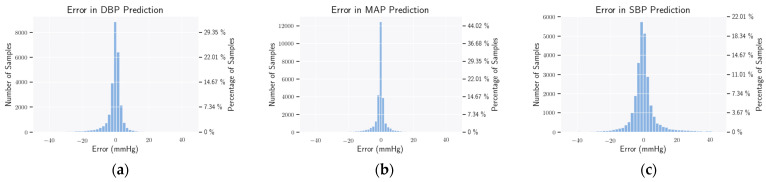
Mean error histogram. Here, we show how the MAE of DBP (**a**), SBP (**b**), and MAP (**c**) of the estimated samples are distributed. From the plots, all errors have a mean of zero and a small standard deviation, except SBP.

It can be observed that for both DBP and MAP, the requirements of the AAMI standard are satisfied quite convincingly. However, for SBP, although the condition of mean error is fulfilled, the value of standard deviation is a bit higher. It may be noted here that other contemporary methods fail to satisfy the AAMI criterion for SBP on the MIMIC-III dataset, as well. The histograms of error for the prediction of DBP, MAP, and SBP are presented in Figure 6. Both for DBP and MAP, these errors have a mean of almost zero with a small value of standard deviation. For SBP, the standard deviations seem more distributed.

### 4.4. Statistical Analysis

**Figure 7:**
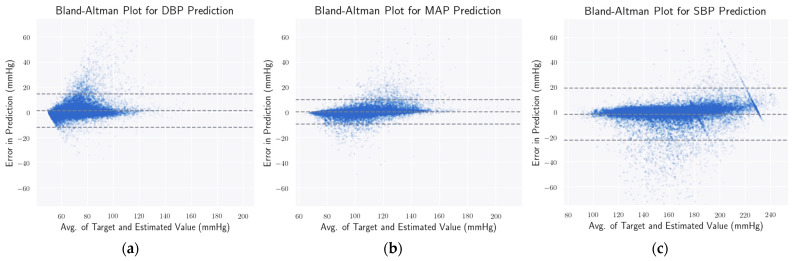
Bland–Altman plot. Here, through the Bland–Altman plots, it is evident that the error of predicting DBP, MAP, and SBP of 95% of the samples lies between [−11.825:15.0637] (**a**), [−9.095:10.357] (**b**), and [−22.531:19.367] (**c**), respectively.

Here, from Figure 7, it is evident that the error of predicting DBP, MAP, and SBP of 95% of the samples lies between [−11.825:15.0637], [−9.095:10.357], and [−22.531:19.367], respectively. This presents the Bland–Altman plots [75] for predicting DBP, MAP, and SBP, respectively. The 95% limits of agreement span the segment from µ − 1.96σ to µ + 1.96σ (shown using dashed lines), where µ and σ are the mean and standard deviation of the distribution, respectively. For DBP, MAP, and SBP, this limit translates to [−11.825:15.064], [−9.095:10.357], and [−22.531:19.367] mmHg, respectively. Though these numbers appear to be overwhelming from the plots from Figure 7, it can be seen that most of the error terms fall within the 5-mmHg range. Nevertheless, all three plots contain a great chunk of outliers, especially the SBP plot (Figure 7c).

**Figure 8:**
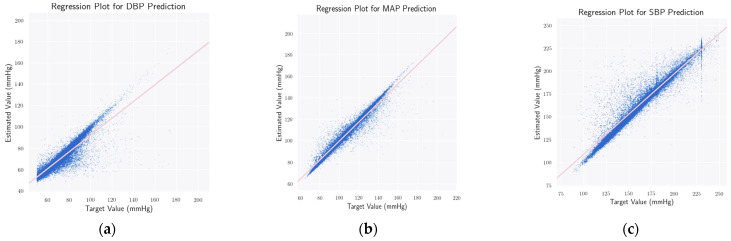
Regression plots for DBP (**a**), MAP (**b**), and SBP (**c**) predictions, respectively. In all three cases, we obtain a *p*-value in the range of *p* < 0.000001, which rejects the null hypothesis and strengthens the statistical significance of our method.

In addition, Figure 8 depicts the regression plots for predicting DBP, MAP, and SBP, respectively. From the plots, the correlation between the predictions and the ground truth is evident. Moreover, the values of the Pearson Correlation Coefficient (PCC) for DBP, MAP, and SBP predictions are 0.894, 0.966, and 0.936, respectively, indicating a strong positive correlation. Furthermore, such high values of PCC on a sample size of 27,260 correspond to (*p* < 0.000001). Stating our null hypothesis to be the lack of any correlation between ground truth and predicted BP values, such a low *p*-value rejects the null hypothesis with a great margin, indicating the statistical significance of our results.

### 4.5. Comparison with the Existing Methods

Despite there being a lot of research endeavors on this topic, there are only some recent works that can be directly compared with our work. However, those recent articles cited our article when it first appeared in the archive. Therefore, this paper is the pioneering work on PPG to ABP synthesis. All other works used the concept of our work while our work was in the review phase for around two years before we withdrew the paper to submit here for fast review. However, to be fair in our comparison to the existing published literature, we have compiled a list of works evaluated on the MIMIC-III dataset with a comparable and sufficiently large number of patients and have presented a comparative analysis in Table 4. Since we have made the pre-processed data and code available on GitHub, several groups have worked on this dataset and managed to publish their article before us. A quote from the Qin et al. [54] paper with proper references, “*Ibtehaz* et al. *76 firstly applies UNet 67, a classic network in the field of medical imaging, in conjunction with the concept of deep supervision, for generating ABP waveform from PPG signal. After that, Athaya et al. 51 did almost the same work as Ibtehaz et al. 76 based on UNet. Mehrabadi et al. 55 apply two networks—LNet and UNet for building ABP waveform predictive model, respectively*.” Therefore, this is the pioneering work in generating ABP signals from PPG alone, which has drawn significant attention from the research community.

## 5. Conclusions

In this study, we tried to estimate Arterial Blood Pressure (ABP) waveforms from Photoplethysmogram (PPG) signals through our proposed PPG2ABP pipeline. PPG2ABP, in its novel approach, implements two 1D-CNN-based segmentation networks in series and aims to gradually estimate the ABP waveforms through approximation and refinement. Unlike the studies which tried to predict only discrete BP parameters, such as SBP, DBP, MAP, and PPG2ABP, it can estimate the ABP waveform itself, which can be used to robustly estimate cardiovascular anomalies from the waveform patterns and the BP parameters. ABP waveforms, which are generally collected invasively, can now be reliably estimated from externally acquired PPG signals. Contrary to some studies, which used ECG signals alongside PPG to estimate ABP, PPG2ABP ignores ECG and manages to reach high performance as well as promises simpler hardware during clinical trials. PPG2ABP is restricted to estimate ABP from only clinical grade finger PPG signals, i.e., it will not perform well with wrist PPG signals collected from wearables, which would be corrupted with severe motion artifacts.

## Figures and Tables

**Table 1:** Statistics of the dataset. Here we present the minimum, maximum, and average values of DBP, MAP, and SBP, respectively. In addition, we also list their standard deviation. It can be observed that SBP values have the highest variance, which makes their prediction the most difficult one, comparatively.

	Min (mmHg)	Max (mmHg)	Mean (mmHg)	Std (mmHg)
DBP	50	165.17	66.14	11.45
MAP	59.96	176.88	90.78	14.15
SBP	71.56	199.99	134.19	22.93

**Table 2:** Evaluation of BHS Standard. Here, we present the criteria used in grading the rank of predictions using the BHS Standard. We also demonstrate how our results compare with the BHS Standard.

		**Cumulative Error Percentage**		
		≤5 mmHg	≤10 mmHg	≤15 mmHg
Our Results	DBP	82.836%	92.157%	95.734%
	MAP	87.381%	95.169%	97.733%
	SBP	70.814%	85.301%	90.921%
BHS	Grade A	60%	85%	95%
	Grade B	50%	75%	90%
	Grade C	40%	65%	85%

**Table 3:** Evaluation of AAMI Standard. Here, we present the criterion used in grading the rank of predictions using the AAMI Standard. We also demonstrate how our results compare with the AAMI Standard.

		ME (mmHg)	STD (mmHg)	Number of Subjects
Our Results	DBP	1.619	6.859	942 [59]
	MAP	0.631	4.962	
	SBP	−1.582	10.688	
AAMI Standard		≤5	≤8	≥85

**Table 4:** Comparison among different approaches. Here, we list the methods that used the MIMIC-III dataset to evaluate their performance. Furthermore, for a fairer comparison, we have only included the methods that consider a significant portion of the dataset. We compare the methods using measures such as Mean Absolute Error (MAE) for predicting DBP, MAP, and SBP, in addition to BHS and AAMI Standard.

Study	Appearing Year	Dataset	Input	Results
Kachuee et al. [58]	2015	MIMIC-III	PPG, ECG	BHS Standard: DBP = Grade B, MAP = Grade C, SBP = Grade D MAE: DBP = 6.34 mmHg, MAP = 7.52 mmHg, SBP = 12.38 mmHg
Kachuee et al. [59]	2016	MIMIC-III	PPG, ECG	BHS Standard: DBP = Grade B, MAP = Grade C, SBP = Grade D AAMI Standard met for DBP, MAP MAE: DBP = 5.35 mmHg, MAP = 5.92 mmHg, SBP = 11.17 mmHg
Mousavi et al. [27]	2019	MIMIC-III	PPG	BHS Standard: DBP = Grade A, MAP = Grade B, SBP = Grade D AAMI Standard met for DBP, MAP
Slapnivcar et al. [19]	2019	MIMIC-III	PPG	MAE: DBP = 9.43 mmHg, SBP = 6.88 mmHg
Athaya et al. [51]	2021	MIMIC-III	PPG	BHS Standard: DBP = Grade A, MAP = Grade A, SBP = Grade A MAE: DBP = 2.17 mmHg, MAP = 1.97 mmHg, SBP = 3.68 mmHg
Harfiya et al. [52]	2021	MIMIC-III	PPG	BHS Standard: DBP = Grade A, MAP = Grade A, SBP = Grade A MAE: DBP = 2.41 mmHg, SBP = 4.05 mmHg
Qin et al. [54]	2021	MIMIC-III	PPG	BHS Standard: DBP = Grade A, MAP = Grade A, SBP = Grade B MAE: DBP = 7.95 mmHg, MAP = 3.83 mmHg, SBP = 4.11 mmHg
Mehrabadi et al. [55]	2022	MIMIC-III	PPG	BHS Standard: DBP = Grade A, MAP = Grade A, SBP = Grade A MAE: DBP = 1.93 mmHg, SBP = 2.29 mmHg
PPG2ABP (Proposed)	2020	MIMIC-III	PPG	BHS Standard: DBP = Grade A, MAP = Grade A, SBP = Grade B AAMI Standard met for: DBP, MAP MAE: DBP = 3.45 mmHg, MAP = 2.31 mmHg, SBP = 5.73 mmHg

Note: Shaded articles used deep learning and have cited our article in the archive. Athaya et al. [51] used the concept of our work but did not cite our work. Please refer to the arxiv version of this article Ibtehaz et al. [76].

## Data Availability

The data used in this experiment along with other relevant documents used to complete this work have been made available or updated in the following GitHub repository [70].

## Supplementary Materials

The following supporting information can be downloaded at: https://www.mdpi.com/article/10.3390/bioengineering9110692/s1, Figure S1: ABP in perspective of the presence of inappropriate signals. For the comparative eminence of skewness in assessing PPG signal quality we have used *S_SQI_* as the grade of PPG signals. It can be observed that as *S_SQI_* increases the overall error of predicting DBP, SBP along with MAE diminishes. Also it should be noted that there were only a few of PPG signals with extremely low *S_SQI_* which was learnt well by the model. Besides, even some good quality PPG signals yielded a low *S_SQI_* score. References [62,77,78] are cited in the supplementary materials.
