# Supplementary material for: PPG2ABP: Translating Photoplethysmogram (PPG) Signals to Arterial Blood Pressure (ABP) Waveforms
Source: Bioengineering (Basel). 2022 Nov 15;9(11):692. doi: 10.3390/bioengineering9110692 (PMC9687508; doi:10.3390/bioengineering9110692)
Supplement: Supplementary file 1 [file bioengineering-09-00692-s001.zip › bioengineering-1954368-supplementary.pdf]

bioengineering

MDPI

Article

PPG2ABP: Translating Photoplethysmogram (PPG) Signals to Arterial Blood Pressure (ABP) Waveforms

Nabil Ibtehaz [1], Sakib Mahmud [2], Muhammad E. H. Chowdhury [2,*], Amith Khandakar [2], Muhammad Salman Khan [2], Mohamed Arselene Ayari [3], Anas M. Tahir [4] and M. Sohel Rahman [5,*]

[1] Department of Computer Science, Purdue University, West Lafayette, IN 47907, USA
[2] Department of Electrical Engineering, Qatar University, Doha 2713, Qatar
[3] College of Engineering, Qatar University, Doha 2713, Qatar
[4] Department of Electrical and Computer Engineering, University of British Columbia, Vancouver, BC V6T 1Z4, Canada; anas5@student.ubc.ca
[5] Department of CSE, BUET, ECE Building, West Palashi, Dhaka 1205, Bangladesh
* Correspondence: mchowdhury@qu.edu.qa, sohel.kcl@gmail.com

Supplementary Materials

Inappropriate Signals

As mentioned earlier, PPG signals get easily corrupted by different types of artefacts. Unfortunately, cleansing PPG signals of these anomalies is no trivial task [77]. Therefore, often a tendency is observed in the existing works, to ignore the noisy PPG signals, which also hinders the computation of handcrafted features. Assessing the quality of PPG signals is also challenging albeit having multiple metrics, due to the inconsistent behavior of the metrics [62]. From the experimental study, it has been established that Skewness based quality index S_{SQI} is the most effective metric in this context [78]. Therefore, we plot the errors in predicting DBP, MAP and SBP against S_{SQI} (Fig. S1). From the plot, it appears that for signals with low S_{SQI} , the error is smaller. However, this is due to the fact that very few signals were in that unfit region and somehow the model managed to learn their patterns with comparative ease. The interesting region is in the middle where the error is the highest. Although this is the acceptable region, the variations therein have made the prediction most difficult. On the contrary, for the excellent region, i.e., with the highest values of S_{SQI} , minimal errors are encountered. Nevertheless, the plots may deceive us as there are some really corrupted signals with a questionably high value of S_{SQI} , and vice versa. Furthermore, the outlier signals in each of the ranges impose the most difficulties.

Mean Error Histogram

The mean error histogram corresponds to the AAMI criterion. From the figures 6, it is again evident that though for DBP and MAP the spread of error is very narrow, it is comparatively outspread for SBP.

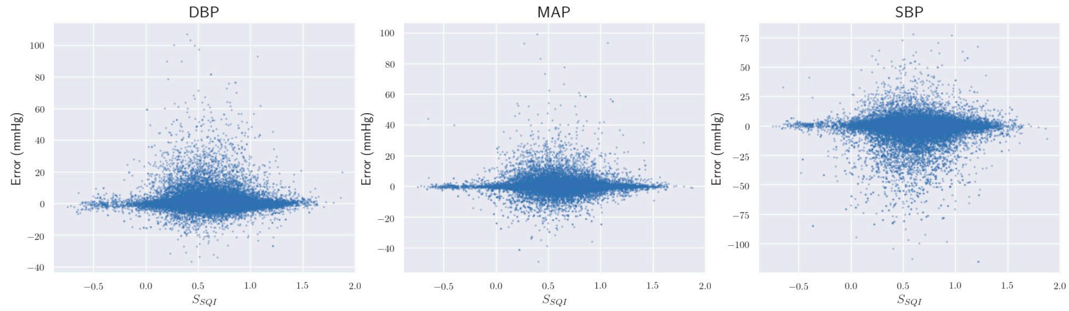

(a) (b) (c)

Figure S1. ABP in perspective of the presence of inappropriate signals. For the comparative eminence of skewness in assessing PPG signal quality we have used S_{SQI} as the grade of PPG signals. It can be observed that as S_{SQI} increases the overall error of predicting DBP, SBP along with MAE diminishes. Also it should be noted that there were only a few of PPG signals with extremely low S_{SQI} which was learnt well by the model. Besides, even some good quality PPG signals yielded a low S_{SQI} score.

Mean Absolute Error Histogram

It can be observed that for both DBP and MAP most of the predictions are covered by the 15-mmHg error threshold, a significant part of which actually fall under 5 mmHg error. For these two, we obtain a grade A score under BHS standard. On the contrary, for SBP though it is apparent from figure 5. that quite a number of test predictions exceed the 15-mmHg error threshold, still it is good enough to achieve the grade B score. It can be observed that PPG2ABP meets the requirements of 5 and 10 mmHg thresholds quite convincingly.
